# 22^nd^ AMN Congress in Bangkok, Thailand – Interview with Prof. Dorel Săndesc

**DOI:** 10.25122/jml-2025-1006

**Published:** 2025-10

**Authors:** Stefana-Andrada Dobran, Alexandra Gherman

**Affiliations:** 1 RoNeuro Institute for Neurological Research and Diagnostic, Cluj-Napoca, Romania; 2 Sociology Department, Babes-Bolyai University, Cluj-Napoca, Romania

**Interviewee: Professor Dorel Săndesc**

**Interviewer: Stefana-Andrada Dobran**

Professor Dorel Săndesc was one of the invited speakers at the 22^nd^ Congress of the Academy for Multidisciplinary Neurotraumatology (AMN), taking place on July 4-5 in Bangkok, Thailand. Here, he addressed strategic and financial considerations behind investing in premium medical therapies or devices, focusing on the need for healthcare leaders to think like CEOs. Moreover, he attended the second debate session, supporting the role of targeted temperature management (TTM) in traumatic brain injury (TBI). Due to his insightful and engaging presentation and argumentation, he was awarded the title of ‘*Highlight of the Congress*’, celebrating his contribution.

At present, he is the general manager at the University County Emergency Hospital Timisoara, Romania, and serves as the President of the Expert Commission of Anesthesia & Intensive Care (ATI) at the Ministry of Health. Prof. Săndesc is Vice-Rector and Head of the Anesthesia and Intensive Care Department at Victor Babes University of Medicine and Pharmacy Timisoara, Romania.

He holds positions as Past-President and Vice-President of the Romanian Society of Anesthesia and Intensive Care and has served in the council of the European Society of Anesthesiology (ESA), the Executive Committee, World Federation Societies of Anesthesiologists (WFSA), and has worked as secretary of state (Deputy minister) in the Ministry of Health in Romania.


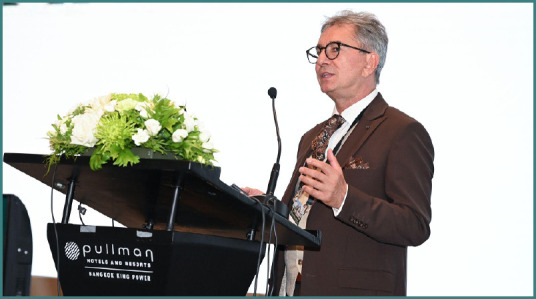


Over his career he has had several contributions to the projects of modernization of Anesthesia and Intensive Care as well as of other specialties, in Romania, and successfully implemented projects and campaigns of social involvement, including the humanitarian project for the development of Anesthesia and Intensive Care Department in Timisoara “Together for Life”, the medical Caravan that has reached several isolated areas in Romania, and the “Vaccination Marathon”, which reached high national impact during the COVID-19 pandemic.


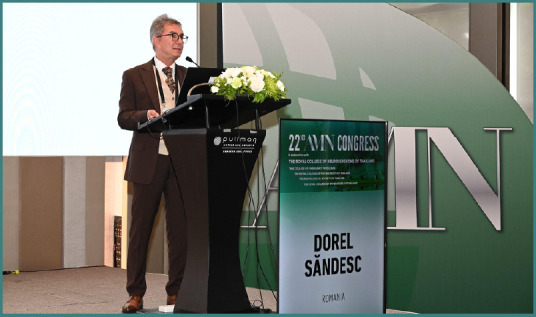


Prof. Săndesc was conferred the title of Knight of the National Order “For Merit” by the President of Romania and is an honorary member of several professional entities. In 2022 he received the “Doctor of the Year” award at the Romanian Healthcare awards as well as “The Campaign of the year” trophy for the Medical Caravans Project.

His scientific activity includes more than 80 ISI articles, several research grants, the role of principal investigator in 22 international clinical trials and three patented inventions.


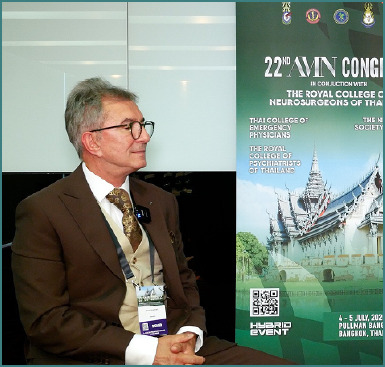


**S.D.: Dear Professor Dorel Sandesc, what is your perspective on the AMN as a global player in the practice and science fields of TBI?**

D.S.: First of all, I'm very happy and honored to be here at this great meeting. I think the AMN touches one of the most important aspects in medical care, medical education, and research. And this is the need for interdisciplinarity. In modern medicine, every specialist goes deep in his [own] language at a level that we sometimes have problems in communication, with communication between specialities. We have a sort of inter-speciality dysphasia. I think this should be addressed and the only way to address it is to come together from all the specialties, all the fields that take care of one pathology - in our situation, neurologic and neurosurgery patients, in order to come to a common language in the interest of our patients.


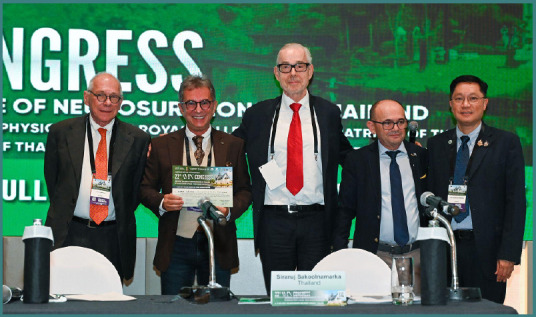


**S.D.: As an anesthesiologist, what is your intake related to the 2025 AMN Congress and what would you foresee as future developments for the complex multidisciplinary domain of TBI?**

D.S.: Neuroanesthesia and neurointensive care is an important, highly specialized part of our profession, of course, because we assure the perioperative management of the patient and the treatment in intensive care units of the most severe cases. I found very interesting topics directly but also indirectly related to our specialties. Here, in this session, in the panel where I have been, we already discussed the subject that is of high interest for me and I think it is not sufficiently explored and managed. This is the burden of survivorship of the patient after the ICU (intensive care unit) – the post-ICU survivorship syndrome that is in its infancy regarding knowledge, education, and research. This is an important problem because patients suddenly have an accident, hemorrhage, and they come into ICU, in coma, they are treated intensively, they are saved, but after that they remain with important sequels and pass through very complex suffering. This syndrome implies physical aspects, like organic deficit, respiratory, neurologic, motor deficit, post-traumatic stress disorder, but also a problem with the necessity to redefine oneself. They enter with one self - for example a guy who is normal, has a lot of quality skills, and after ICU, after they survived, they have deficits and it's very difficult to understand and to adapt. Until now we did not address this subject. Sometimes we feel angry, frustrated that our patients that we saved, do not want to come to see us again. And we think they are not grateful, but in fact they have this syndrome. Because for them, ICU is a place where they have lost a lot of qualities and skills. They have a lot of deficiencies. So we have to address this subject. We have to create scales to evaluate the severity of this syndrome and to find ways to help them cope with a new identity and realize the move-on concept, to realize, in fact, they are saved from a very critical situation. They could be dead, but they survived and they could live with the problems they have, as other people do, and they can be happy. And I'm happy that we have discussed this with other colleagues. We need experts from statistics, from neurology, from different fields in order to address this very sensitive problem that tends to be one of the major challenges in intensive care units and in hospitals in general.

**S.D.: How would you envision your future collaboration within the AMN academic environment?**

D.S.: I am impressed by the evolution of this organization, the fact that here in this organization are the biggest experts from different fields, and I am very much interested to continue to cooperate. I am very much interested in developing AMN presence in Romania, for example, and why not, to start a working group on this subject – post-ICU survivorship syndrome. So, I hope we will stay together and work together for the benefit of our patients.

